# Supplementary material for: Transcriptional response of a target plant to benzoxazinoid and diterpene allelochemicals highlights commonalities in detoxification
Source: BMC Plant Biol. 2022 Aug 17;22:402. doi: 10.1186/s12870-022-03780-w (PMC9382751; doi:10.1186/s12870-022-03780-w)
Supplement: Supplementary file 1 — Additional file 1: Supplemental Figure 1. Momilactone B dose response. (A) Growthphenotype of A. thaliana seedlings grown on ½ MS-agar with varyingconcentrations of momilactone B. Scale bar = 1 cm. (B) Primary root lengthplotted as relative percentage of growth compared to the control sample. Supplemental Figure 2.Overrepresentation analysis of down-regulated genes. Lollipop plot of geneontology (GO) terms of genes included in clusters A5 and A10 (APO) or not partof a cluster (“Not correlated”, momilactone B). Genes with a negative log foldchange and an adjusted p-value < 0.01 were included in an overrepresentationanalysis of GO terms. Orange bars indicate the number of genes belonging to aparticular GO-term relative to the total number of genes belonging to the term,blue bars indicate number of genes belonging to the GO-term compared to thetotal number of genes in the genome. Circle fill color indicates the p-value ofthe hypergeometric test, adjusted for multiple comparisons using the method ofBenjamini-Hochberg [1]. SupplementalFigure 3. Momilactone content in Oryza sativa cv. Kitaake (Rice) andEchinochloa crus-galli (ECG). Momilactone A and B in MeOH extracts from rootsof three week old rice and E. crus-galli were measured by LCMS. Boxplotssummarizing 18 replicates are shown, boxes indicate 1st to 3rd quartile,horizontal line indicates the median, whiskers extend to quartile1-1,5*IQR andquartile3+1,5*IQR. Supplemental Table 1.RNA-seq mapping statistics. SupplementalTable 2. Differentially expressed CYP45s. [file 12870_2022_3780_MOESM1_ESM.pdf]

Supplemental Material to

Transcriptional response of a target plant to benzoxazinoid and diterpene allelochemicals highlights commonalities in detoxification

Eva Knoch, Judit Kovács, Sebastian Deiber, Reshi Shanmuganathan, Núria Serra Serra, Claude Becker, Niklas Schandry

This file contains:

- Supplemental Figures 1-3
- Supplemental Tables 1-2
- Supplemental References

Supplemental Figure 1

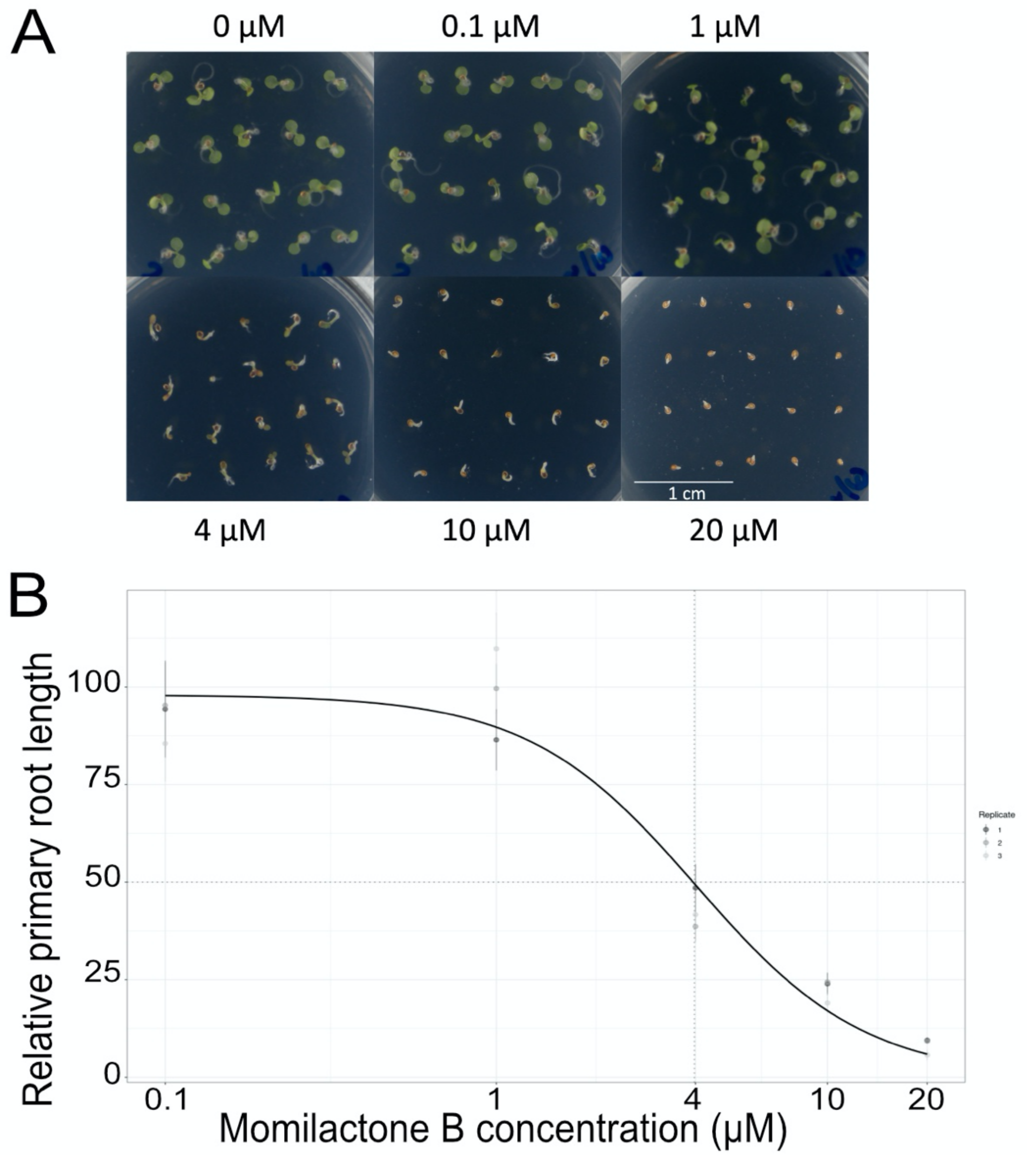

Supplemental Figure 1. Momilactone B dose response. **(A)** Growth phenotype of *A. thaliana* seedlings grown on ½ MS-agar with varying concentrations of momilactone B. Scale bar = 1 cm. **(B)** Primary root length plotted as relative percentage of growth compared to the control sample.

Supplemental Figure 2

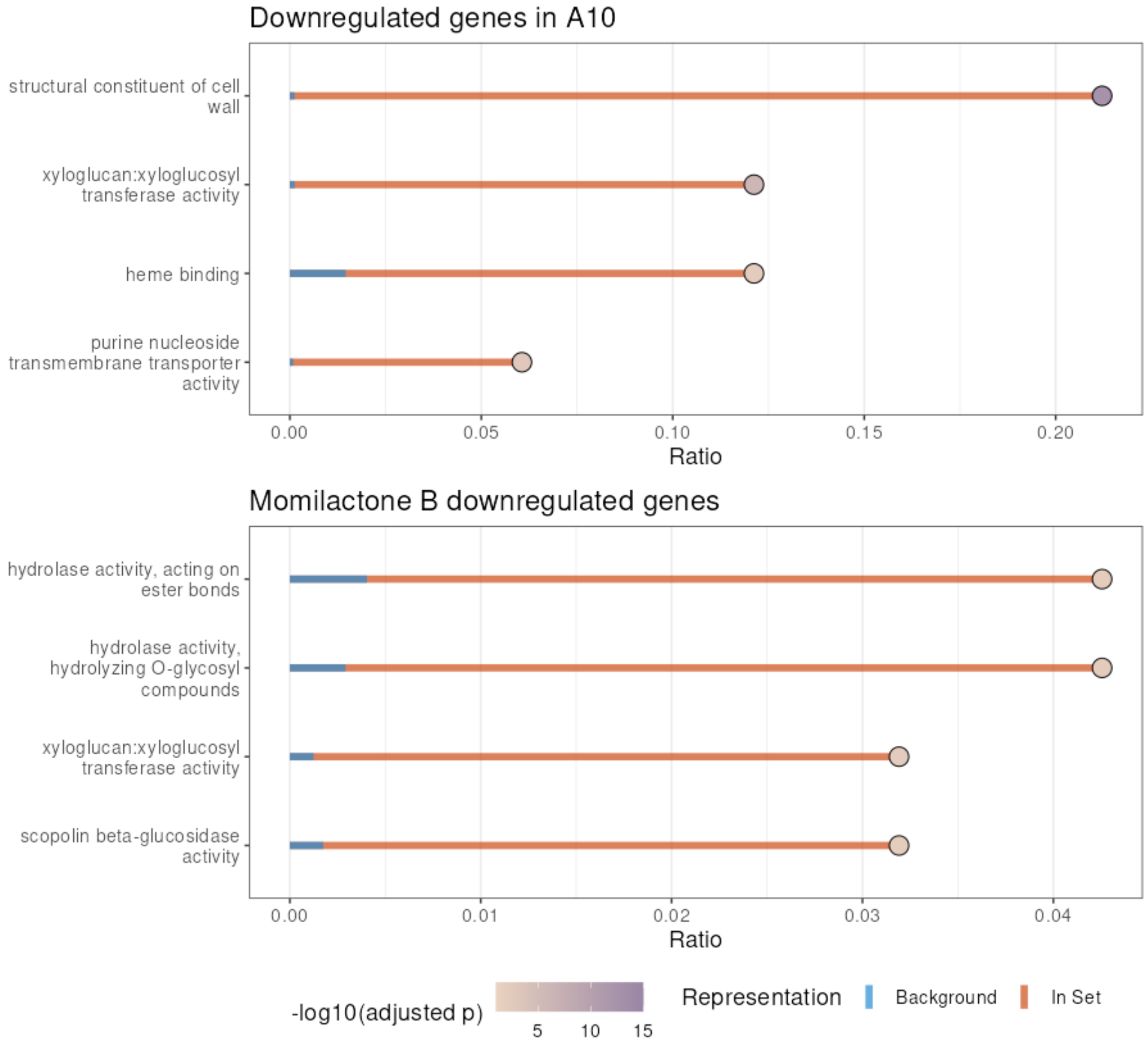

Supplemental Figure 2: Overrepresentation analysis of down-regulated genes. Lollipop plot of gene ontology (GO) terms of genes included in clusters A5 and A10 (APO) or not part of a cluster ("Not correlated", momilactone B). Genes with a negative log fold change and an adjusted p-value < 0.01 were included in an overrepresentation analysis of GO terms. Orange bars indicate the number of genes belonging to a particular GO-term relative to the total number of genes belonging to the term, blue bars indicate number of genes belonging to the GO-term compared to the total number of genes in the genome. Circle fill color indicates the p-value of the hypergeometric test, adjusted for multiple comparisons using the method of Benjamini-Hochberg [1].

Supplemental Figure 3

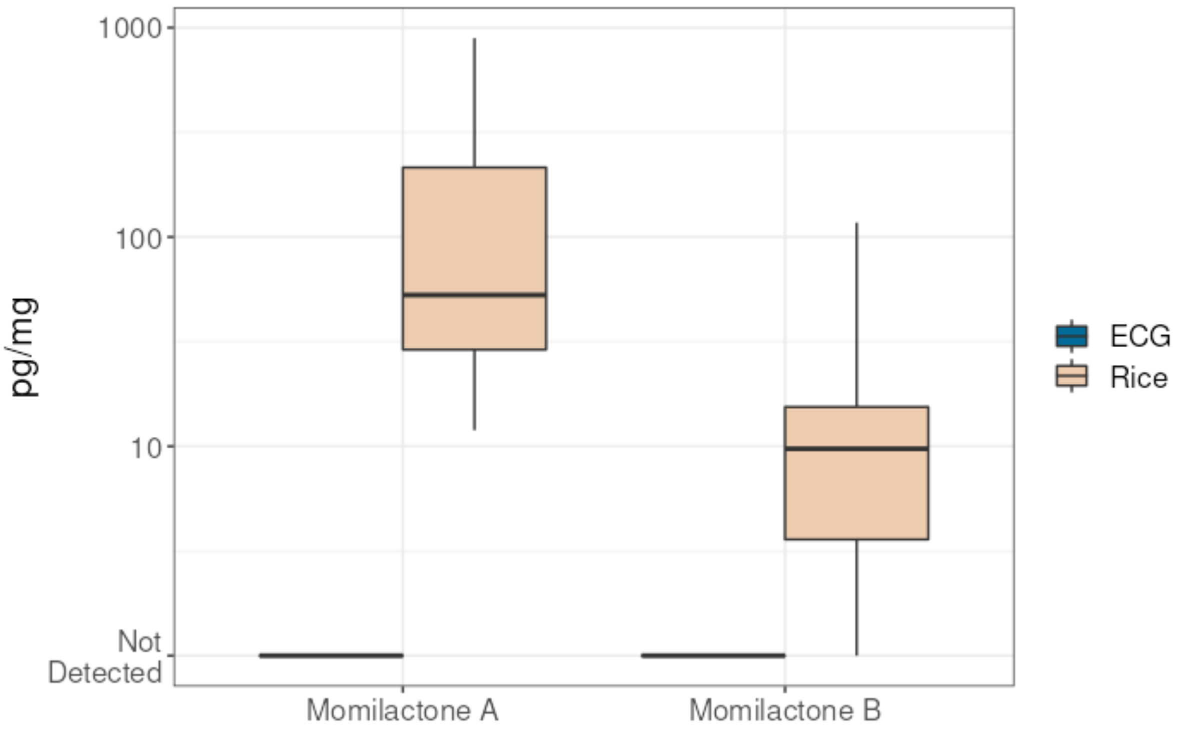

Supplemental Figure 3. Momilactone content in Oryza sativa cv. Kitaake (Rice) and Echinochloa crus-galli (ECG). Momilactone A and B in MeOH extracts from roots of three week old rice and *E. crus-galli* were measured by LCMS. Boxplots summarizing 18 replicates are shown, boxes indicate 1st to 3rd quartile, horizontal line indicates the median, whiskers extend to quartile1-1,5*IQR and quartile3+1,5*IQR

Supplemental Table 1

Sample	Experiment	Input	Unique mapped	% Unique mapped	Multimapped	% Multimapped
APO_1_A	APO	33468367	32635808	97.51%	598071	1.79%
APO_1_B	APO	13303280	12958891	97.41%	253520	1.91%
APO_1_C	APO	13207791	12886546	97.57%	226331	1.71%
APO_1_D	APO	13831522	13492279	97.55%	241370	1.75%
APO_24_A	APO	12077780	11739344	97.20%	237876	1.97%
APO_24_B	APO	12844730	12472823	97.10%	285025	2.22%
APO_24_C	APO	13157352	12804481	97.32%	267380	2.03%
APO_24_D	APO	12244253	11917106	97.33%	244987	2.00%
APO_6_A	APO	15201412	14850778	97.69%	247448	1.63%
APO_6_B	APO	14864098	14484105	97.44%	288045	1.94%
APO_6_C	APO	12675530	12360557	97.52%	234350	1.85%
APO_6_D	APO	14154148	13800289	97.50%	244854	1.73%
DMSO_0_A	APO	18604718	18079070	97.17%	379269	2.04%
DMSO_0_B	APO	15526404	15082736	97.14%	345099	2.22%
DMSO_0_C	APO	14478446	14089113	97.31%	282423	1.95%
DMSO_0_D	APO	12652797	12331808	97.46%	234548	1.85%
DMSO_1_A	APO	21520940	20919612	97.21%	450808	2.09%
DMSO_1_B	APO	15108745	14695726	97.27%	313665	2.08%
DMSO_1_C	APO	12995297	12647493	97.32%	250166	1.93%
DMSO_1_D	APO	11787630	11471172	97.32%	226384	1.92%
DMSO_24_A	APO	14126302	13711086	97.06%	322305	2.28%
DMSO_24_B	APO	15213373	14818120	97.40%	299031	1.97%
DMSO_24_C	APO	12714549	12350707	97.14%	278302	2.19%
DMSO_24_D	APO	12464661	12107701	97.14%	253034	2.03%
DMSO_6_A	APO	14850544	13721149	92.39%	817986	5.51%
DMSO_6_B	APO	15272280	14626289	95.77%	323714	2.12%
DMSO_6_C	APO	13155897	12451840	94.65%	212284	1.61%
DMSO_6_D	APO	13227781	12609698	95.33%	258050	1.95%
DMSO_0h_1	Momilactone B	14382197	1123045	7.81%	81329	0.57%
DMSO_0h_2	Momilactone B	3958297	3469868	87.66%	330821	8.36%
DMSO_0h_3	Momilactone B	1417280	780139	55.04%	365062	25.76%
DMSO_0h_4	Momilactone B	10901026	9199207	84.39%	1481899	13.59%
DMSO_1h_1	Momilactone B	10194188	8774305	86.07%	978795	9.60%
DMSO_1h_2	Momilactone B	14674393	13351529	90.99%	945121	6.44%
DMSO_1h_3	Momilactone B	18902989	14669222	77.60%	2630303	13.91%
DMSO_1h_4	Momilactone B	9533524	8233576	86.36%	843176	8.84%
DMSO_24h_1	Momilactone B	22178704	20128771	90.76%	1267084	5.71%
DMSO_24h_2	Momilactone B	24088746	20114579	83.50%	3123586	12.97%
DMSO_24h_3	Momilactone B	20218885	17022949	84.19%	2460316	12.17%
DMSO_24h_4	Momilactone B	23610363	21046925	89.14%	1541774	6.53%
DMSO_6h_2	Momilactone B	21041416	18199018	86.49%	1376492	6.54%
DMSO_6h_3	Momilactone B	22702394	19229227	84.70%	2805472	12.36%
DMSO_6h_4	Momilactone B	24868740	18401963	74.00%	5853970	23.54%
MomB_1h_1	Momilactone B	19396480	17997922	92.79%	1152979	5.94%
MomB_1h_2	Momilactone B	15280820	12593194	82.41%	2351135	15.39%
MomB_1h_3	Momilactone B	24227029	21875372	90.29%	1885258	7.78%
MomB_1h_4	Momilactone B	19499176	15968805	81.89%	3216737	16.50%
MomB_24h_1	Momilactone B	29161388	24142676	82.79%	4103996	14.07%
MomB_24h_2	Momilactone B	22966052	19388213	84.42%	2947973	12.84%
MomB_24h_3	Momilactone B	15904026	14125386	88.82%	1309675	8.23%
MomB_24h_4	Momilactone B	16506046	14677116	88.92%	1517243	9.19%
MomB_6h_1	Momilactone B	16784399	15841123	94.38%	781481	4.66%
MomB_6h_2	Momilactone B	17026600	15717196	92.31%	1053980	6.19%
MomB_6h_3	Momilactone B	24497494	21802140	89.00%	2115931	8.64%
MomB_6h_4	Momilactone B	13566887	11009230	81.15%	2273091	16.75%

Supplemental Table 1. RNA-seq mapping statistics.

Supplemental Table 2

Supplemental Table 2: Differentially expressed CYP450s

CYP	GeneId	Also found in
APO		
CYP705A20	AT3G20110	-
CYP705A5	AT5G47990	-
CYP708A2	AT5G48000	-
CYP71B15	AT3G26830	Brazier-Hicks et al.
CYP72A13	AT3G14660	Brazier-Hicks et al.
CYP72A15	AT3G14690	Brazier-Hicks et al.
CYP72A8	AT3G14620	Brazier-Hicks et al. & Baerson et al.
CYP75B1	AT5G07990	-
CYP81D11	AT3G28740	Brazier-Hicks et al. & Baerson et al.
CYP81D8	AT4G37370	Brazier-Hicks et al. & Baerson et al.
CYP81H1	AT4G37310	Brazier-Hicks et al.
CYP86A8	AT2G45970	-
CYP86B1	AT5G23190	Brazier-Hicks et al.
CYP86B2	AT5G08250	Brazier-Hicks et al.
CYP89A2	AT1G64900	Brazier-Hicks et al.
CYP89A5	AT1G64950	-
Momilactone B		
CYP706A1	AT4G22690	-
CYP706A2	AT4G22710	-
CYP707A3	AT5G45340	-
CYP710A1	AT2G34500	Brazier-Hicks et al.
CYP714A1	AT5G24910	-
CYP71A12	AT2G30750	-
CYP71B15	AT3G26830	Brazier-Hicks et al.
CYP72A8	AT3G14620	Brazier-Hicks et al. & Baerson et al.
CYP76C2	AT2G45570	-
CYP79B2	AT4G39950	-
CYP81D11	AT3G28740	Brazier-Hicks et al. & Baerson et al.
CYP81D8	AT4G37370	Brazier-Hicks et al. & Baerson et al.
CYP81F2	AT5G57220	Brazier-Hicks et al. & Baerson et al.
CYP81F3	AT4G37400	-
CYP83B1	AT4G31500	-
CYP89A5	AT1G64950	-

Supplemental Table 2. Differentially expressed Cytochrome P450 oxidases (CYPs). All CYPs that were significantly ($p < 0.01$) differentially expressed at any timepoint are listed. The first column provides the CYP name, the second column shows the corresponding Col-0 locus identifier, the last column indicates if the particular genes was also found as differentially expressed upon femclorin [2] or BOA treatment [3]. Bold type highlights CYPs that were differentially expressed upon both APO and momilactone treatment.

Supplemental references

1. Benjamini Y, Hochberg Y. Controlling the False Discovery Rate: A Practical and Powerful Approach to Multiple Testing. J R Stat Soc Ser B Methodol. 1995;57:289–300.
2. Brazier-Hicks M, Gershater M, Dixon D, Edwards R. Substrate specificity and safener inducibility of the plant UDP-glucose-dependent family 1 glycosyltransferase super-family. Plant Biotechnol J. 2018;16:337–48.
3. Baerson SR, Sánchez-Moreiras A, Pedrol-Bonjoch N, Schulz M, Kagan IA, Agarwal AK, et al. Detoxification and transcriptome response in Arabidopsis seedlings exposed to the allelochemical benzoxazolin-2(3H)-one. J Biol Chem. 2005;280:21867–81.
